# Social Support, Isolation, and Cognitive Health among LGBTQ+ Older Adults in the United States

**DOI:** 10.1002/alz.087479

**Published:** 2025-01-09

**Authors:** Nik M. Lampe, Tara McKay

**Affiliations:** ^1^ University of South Florida, Tampa, FL USA; ^2^ Vanderbilt University, Nashville, TN USA

## Abstract

**Background:**

Lack of social support and social isolation are risk factors for poor cognitive health. Lesbian, gay, bisexual, transgender, and queer (LGBTQ) older adults consistently report higher levels of isolation and less social support in population studies. In this study, we test the effects of support for LGBTQ+ identity and feelings of social isolation on subjective cognitive health in a cohort of LGBTQ+ older Americans.

**Method:**

We use Wave 1 data from the Vanderbilt University Social Networks, Aging, and Policy Study (VUSNAPS). VUSNAPS recruited 1,256 LGBTQ+ adults aged 50+ in four US states between 2020‐2021. Subjective cognitive problems were measured using a 15‐item scale capturing problems with memory, cognition, and functioning. Chi‐square tests and logistic and Poisson regressions were used to test associations between levels of support from family, coworkers, neighbors, and number of days feeling isolated in the last 7 days with the number of cognitive difficulties reported and reporting >4 cognitive difficulties. Analyses control for gender, race, education, employment status, personal income, age, and married/partnered status.

**Result:**

About 11% of respondents reported having >4 cognitive difficulties. Compared to respondents reporting <4 cognitive difficulties, respondents reporting >4 cognitive problems were more likely to be bisexual (16.8 vs 10.6%, *p*<.05), report personal income lower than US median personal income (16.2% vs 7.5%, *p*<.001), and be working less than full time (13.6 vs 7.3%, *p*<.001). Respondents with LGBTQ+ supportive families and coworkers reported significantly fewer cognitive problems (IRR = 0.698, *p*<.001; IRR = 0.846, *p*<0.1), and those with supportive families were 43.2% less likely to report >4 cognitive problems (*p*<.01). Presence of unsupportive families, coworkers, neighbors, and friends were significantly correlated with feelings of social isolation (*p*<.01 for each). One third of respondents (35.0%) reported feeling isolated ≥4 of the last 7 days. Respondents who reported feeling isolated ≥4 days were two‐and‐a‐half times more likely to report ≥4 cognitive problems (OR = 2.67; *p*<.001).

**Conclusion:**

We find associations between subjective cognitive health and LGBTQ‐specific social support in a large sample of older LGBTQ+ adults. Availability of social support and risk of social isolation should be assessed when administering cognitive screenings for LGBTQ+ older patients.

